# Saikosaponin A and D Inhibit Adipogenesis via the AMPK and MAPK Signaling Pathways in 3T3-L1 Adipocytes

**DOI:** 10.3390/ijms222111409

**Published:** 2021-10-22

**Authors:** Sung Ho Lim, Ho Seon Lee, Hyo-Kyung Han, Chang-Ik Choi

**Affiliations:** 1 Integrated Research Institute for Drug Development, College of Pharmacy, Dongguk University-Seoul, Goyang 10326, Korea; 93sho617@naver.com (S.H.L.); ghtjsrhtn@naver.com (H.S.L.); 2 BK21 FOUR Team and Integrated Research Institute for Drug Development, College of Pharmacy, Dongguk University-Seoul, Goyang 10326, Korea; hkhan@dongguk.edu

**Keywords:** saikosaponin, 3T3-L1 cells, adipogenesis, AMPK pathway, MAPK pathway

## Abstract

Obesity is a lipid metabolism disorder caused by genetic, medicinal, nutritional, and other environmental factors. It is characterized by a complex condition of excess lipid accumulation in adipocytes. Adipogenesis is a differentiation process that converts preadipocytes into mature adipocytes and contributes to excessive fat deposition. Saikosaponin A (SSA) and saikosaponin D (SSD) are triterpenoid saponins separated from the root of the Bupleurum chinensis, which has long been used to treat inflammation, fever, and liver diseases. However, the effects of these constituents on lipid accumulation and obesity are poorly understood. We investigated the anti-obesity effects of SSA and SSD in mouse 3T3-L1 adipocytes. The MTT assay was performed to measure cell viability, and Oil Red O staining was conducted to determine lipid accumulation. Various adipogenic transcription factors were evaluated at the protein and mRNA levels by Western blot assay and quantitative reverse transcription polymerase chain reaction (qRT-PCR). Here, we showed that SSA and SSD significantly inhibited lipid accumulation without affecting cell viability within the range of the tested concentrations (0.938–15 µM). SSA and SSD also dose-dependently suppressed the expression of peroxisome proliferator-activated receptor gamma (PPARγ), CCAAT/enhancer binding protein alpha (C/EBPα), sterol regulatory element binding protein-1c (SREBP-1c), and adiponectin. Furthermore, the decrease of these transcriptional factors resulted in the repressed expression of several lipogenic genes including fatty acid binding protein (FABP4), fatty acid synthase (FAS), and lipoprotein lipase (LPL). In addition, SSA and SSD enhanced the phosphorylation of adenosine monophosphate-activated protein kinase (AMPK) and its substrate, acetyl-CoA carboxylase (ACC), and inhibited the phosphorylation of extracellular-regulated kinase 1/2 (ERK1/2) and p38, but not c-Jun-N-terminal kinase (JNK). These results suggest that SSA and SSD inhibit adipogenesis through the AMPK or mitogen-activated protein kinase (MAPK) pathways in the early stages of adipocyte differentiation. This is the first study on the anti-adipogenic effects of SSA and SSD, and further research in animals and humans is necessary to confirm the potential of saikosaponins as therapeutic agents for obesity.

## 1. Introduction

Obesity is characterized by the excess storage of fat due to an imbalance between energy intake and expenditure [1]. There is an increase in the number of differentiated adipocytes, which are regulated by genetic and environmental factors [2]. This complex condition is closely associated with the occurrence of metabolic diseases and cardiovascular diseases including type 2 diabetes mellitus, dyslipidemia, coronary heart disease, atherosclerosis, hypertension, and stroke; therefore, the treatment and prevention of obesity are very important [3]. According to the 2020 World Health Organization report, 39% and 13% of adults aged 18 years and older are overweight and obese, respectively. Additionally, the worldwide prevalence of obesity nearly tripled between 1975 and 2020 [4]. Referring to this global phenomenon, a new word, “globesity”, was created [5].

Adipogenesis is a complex multi-step process by which preadipocytes are converted into differentiated adipocytes [6]. This differentiation process involves many key transcriptional factors including cytosine-cytosine-adenosine-adenosine-thymidine (CCAAT)/enhancer-binding protein (C/EBP) family members (C/EBP-α, C/EBP-β and C/EBP δ) and peroxisome proliferator-activated receptor (PPAR) family members (PPARα, PPARβ/δ, and PPARγ) [7]. Sterol regulatory element binding protein-1c (SREBP-1c), also known as adipocyte differentiation-dependent factor 1 (ADD1), is regulated by C/EBP factors. In addition, SREBP-1c contributes to the expression of PPARγ and the production of endogenous PPARγ ligands [8]. Subsequently, these key transcription factors can activate the expression of a variety of adipocyte-specific genes, which are associated with lipogenesis and adipogenesis, including adiponectin, fatty acid binding protein (FABP4), fatty acid synthase (FAS), and lipoprotein lipase (LPL) [9]. Therefore, reducing the expression and activity of these key transcription factors associated with adipogenesis has been suggested as an effective strategy for the treatment of obesity.

High-cost synthetic anti-obesity drugs for the treatment of obesity, such as orlistat, lorcaserin, naltrexone, and liraglutide, have shown successful weight loss relative to placebo [10]. However, various adverse events have been reported for them, including cardiovascular toxic effects, hallucinations, headache, anxiety, and severe hepatic adverse events. To overcome these problems, studies on the anti-obesity effects of various natural bioactive compounds quercetin, kaempferol, and gallic acid have been conducted. These bioactive components reduced adipose tissue mass by promoting lipolysis, inhibiting adipose tissue development, and preadipocyte differentiation [11]. Further research in this field will be an excellent alternative treatment strategy for developing effective and safe anti-obesity drugs in the future.

Saponins are principally produced by plants, but they have also been isolated from some bacteria and lower marine animals [12]. Their structure consists of a sugar moiety linked to a hydrophobic aglycone called sapogenin. The sugar moiety may contain glucose, galactose, rhamnose, methylpentose, glucuronic acid, or xylose, whereas the aglycone portion may be a steroid or a triterpene [13]. Steroidal saponins are abundant in monocotyledons and triterpenoid saponins are predominantly contained in dicotyledons. These compounds have many pharmacological effects, such as antifungal, insecticidal, antihelmintic, cytotoxic, anti-inflammatory, immunostimulant, hypocholesterolemic and hypoglycemic properties [14]. Furthermore, the potential anti-obesity activity and mechanisms of various saponins have been investigated over the last few decades. As a result, saponin-rich extracts and saponins have been demonstrated to inhibit pancreatic lipase and modulate adipogenesis or regulate appetite [15].

*Radix Bupleuri* (Shiho in Korean, Saiko in Japanese, Chaihu in Chinese) is derived from the roots of *Bulpeurum chinense* (B. chinensis) DC. and *B. scorzonerifolium* Wild. Indeed, many other *Bupleurum* species are used such as *Radix Bupleuri* in East Asia, including *B. falcatum* L., which is recorded in the Korean and Japanese Pharmacopeia, and *B. yinchowense* Shan and Li., which is listed in the provincial Pharmacopeia of China [16]. Furthermore, it has many valuable and important pharmacology activities, such as anti-inflammatory, anticancer, antiviral, hepatoprotective, and immunoregulation effects [17]. About 50 species from *Radix Bupleuri* (*B. falcatum*, *B. chinense*, *B. scorzonerifolium*, *B. polyclonum*, *B. kunmingense*, etc.) have been investigated chemically, resulting in the isolation of approximately 120 derivatives of saikosaponins, more than 50 lignans, as well as a number of coumarins, flavonoids, polyacetylenes, polysaccharides, sterols, phenylpropanoids, and organic acids [18].

Over 100 triterpenoid saponins have been isolated from *Radix Bupleuri* in recent years. Among them, saikosaponin A (SSA) and saikosaponin D (SSD), major bioactive compounds, have been shown to have various pharmacological activities such as anti-proliferation, anti-inflammatory, anti-convulsant, anticancer, antiviral, neuromodulation, and immunoregulation effects [19]. Saikosaponins have shown potential as promising new therapeutic agents for the treatment of obesity through 5-hydroxytryptamine 2C receptor agonistic activity and inhibition of inflammation-related genes in 3T3-L1 hypertrophied adipocytes [20,21]. However, studies on the adipogenesis-specific target identification and underlying mechanisms involved in anti-obesity activity of saikosaponins have not yet been reported. Therefore, we verified the adipogenesis inhibitory effect and molecular mechanisms of SSA and SSD using the 3T3-L1 adipocytes.

## 2. Results

### 2.1. Effect of SSA and SSD on Cell Viability

**Figure 1:**
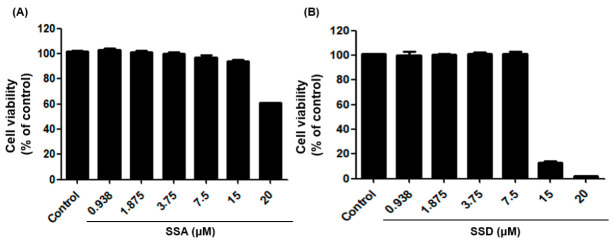
Effect of saikosaponin A (SSA, **A**) and saikosaponin D (SSD, **B**) treatment on cell viability in 3T3-L1 cells for 48 h. The cell viability was assessed by MTT assay. Data are expressed as a percentage of control (0.5% DMSO) value of triplicate experiments.

The MTT assay was performed on 3T3-L1 pre-adipocytes to measure the effects of SSA and SSD on cell viability. First, 3T3-L1 preadipocytes were cultured for 24 h and then incubated for 48 h with compounds up to a maximum concentration of 20 μM. Our results showed that SSA did not affect cell viability up to 15 μM. However, treatment with 15 μM of SSD induced significant cell damage and decreased cell viability (Figure 1). Based on these results, we used concentrations in the range of 1.875–15 µM for SSA and 0.938–7.5 µM for SSD for further studies.

### 2.2. Effect of SSA and SSD on Lipid Accumulation

**Figure 2:**
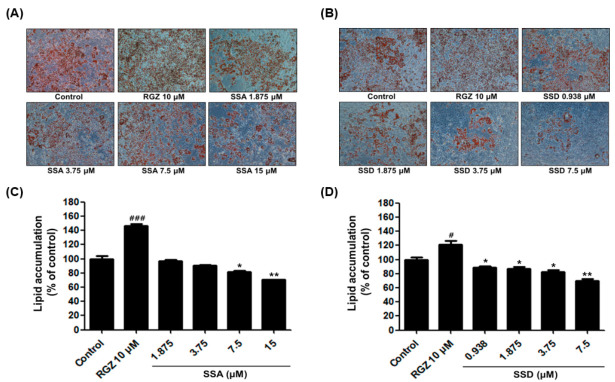
Effect of SSA and SSD on lipid accumulation in 3T3-L1 cells. (**A**,**B**) Representative cell images were captured at 100 × magnification. (**C**,**D**) The lipid droplets were stained Oil Red O solution and extracted using isopropanol. Then, they were quantified using a microplate reader at the wavelength of 520 nm. Data are presented as a percentage of control (0.5% DMSO) and RGZ (rosiglitazone) was used as a positive control for this experiment. Results are mean ± standard error of the mean (SEM, *n* = 3); * *p* < 0.05, ** *p* < 0.01, # *p* < 0.05, ### *p* < 0.001, compared with control. #: increased compared to control, *: decreased compared to control.

Lipid accumulation is a crucial indicator of adipogenesis. To investigate the effect of SSA and SSD on lipid accumulation, 3T3-L1 preadipocytes were induced to differentiate with differentiation initiation medium (DIM), differentiation progression medium (DPM), and post-differentiation medium (PDM) for 8 days. After differentiation, cells were fixed in 10% formalin and stained with Oil Red O solution. As shown by microscopy (Figure 2), SSA and SSD inhibited lipid droplets in fully differentiated cells. To determine the cellular lipid amount, stained cells were eluted with isopropanol, and spectrophotometrically analyzed at 520 nm using a microplate reader. Our results showed that SSA and SSD dose-dependently downregulated lipid accumulation compared with the controls. Our data suggest that SSA and SSD significantly suppressed the differentiation of 3T3-L1 adipocytes.

### 2.3. Effect of SSA and SSD on the Expressions of PPARγ and C/EBPα

**Figure 3:**
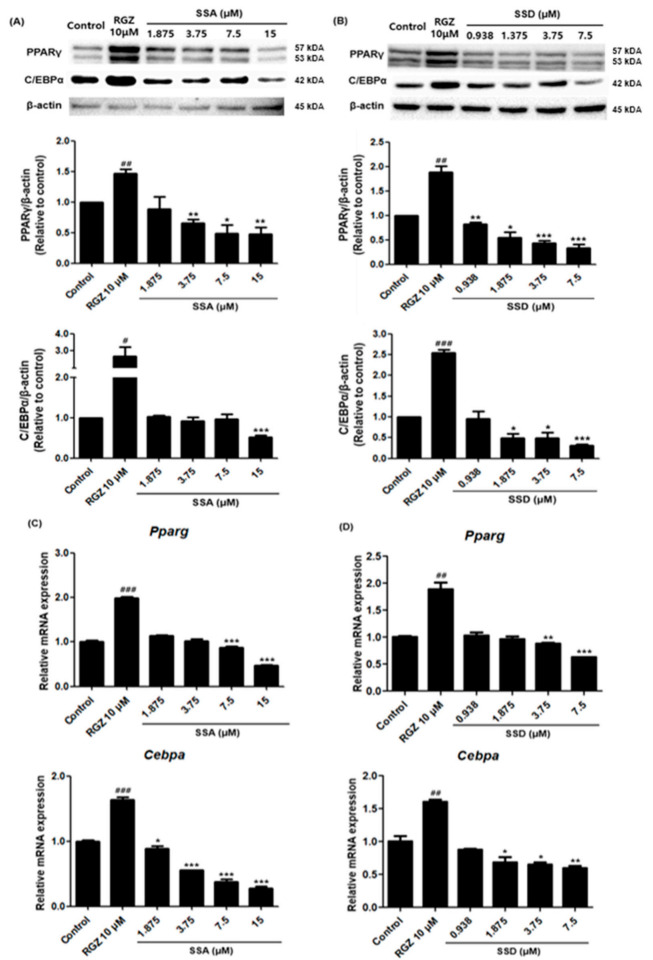
Effect of SSA and SSD on the protein and mRNA expression levels of PPARγ and C/EBPα in 3T3-L1 cells. Cells were seeded in a 6-well plate and stimulated to differentiate with DIM, DPM and PDM in the presence or absence of SSA and SSD (0.938, 1.875, 3.75, 7.5 and 15 µM) for 8 days. (**A**,**B**) The protein levels of PPARγ and C/EBPα were analyzed using Western blot with specific antibodies. (**C**,**D**) Relative mRNA expression levels of *Pparg* and *Cebpa* were measured by quantitative real-time PCR. Results are shown as a relative of control (0.5% DMSO) values and RGZ (rosiglitazone) was used as a positive control for these experiments. The β-actin was used as a loading control and the *Gapdh* was used as a house keeping gene. Target gene mRNA levels were normalized to *Gapdh* using the 2-ΔΔ Ct method. Data are mean ± standard error of the mean (SEM) of three independent experiments. * *p* < 0.05, ** *p* < 0.01, *** *p* < 0.001, # *p*< 0.05, ## *p* < 0.01, ### *p* < 0.001 compared with control, #: increased compared to control, *: decreased compared to control.

Differentiation processes related to lipid accumulation were induced by increased levels of several adipogenic transcription factors. To determine the anti-adipogenic effects of SSA and SSD in 3T3-L1 cells, the expressions of adipogenic-specific factors including PPARγ and C/EBPα were examined. As shown in Figure 3, the protein levels of PPARγ and C/EBPα decreased after treatment with the highest concentrations of SSA and SSD. Particularly, SSD suppressed these key transcription factors in a dose-dependent manner. In addition, treatment with these components also inhibited the mRNA expressions of PPARγ and C/EBPα. The downregulation effects of SSA and SSD were slight at 0.938 µM and significant at 7.5 µM. These results show that treatment with SSA and SSD reduced the expressions of PPARγ and C/EBPα, known as master transcription regulators of adipogenesis, which are expressed early in the adipocyte differentiation of 3T3-L1 cells.

### 2.4. Effects of SSA and SSD on the Expressions of Adipogenesis-Related Genes

**Figure 4:**
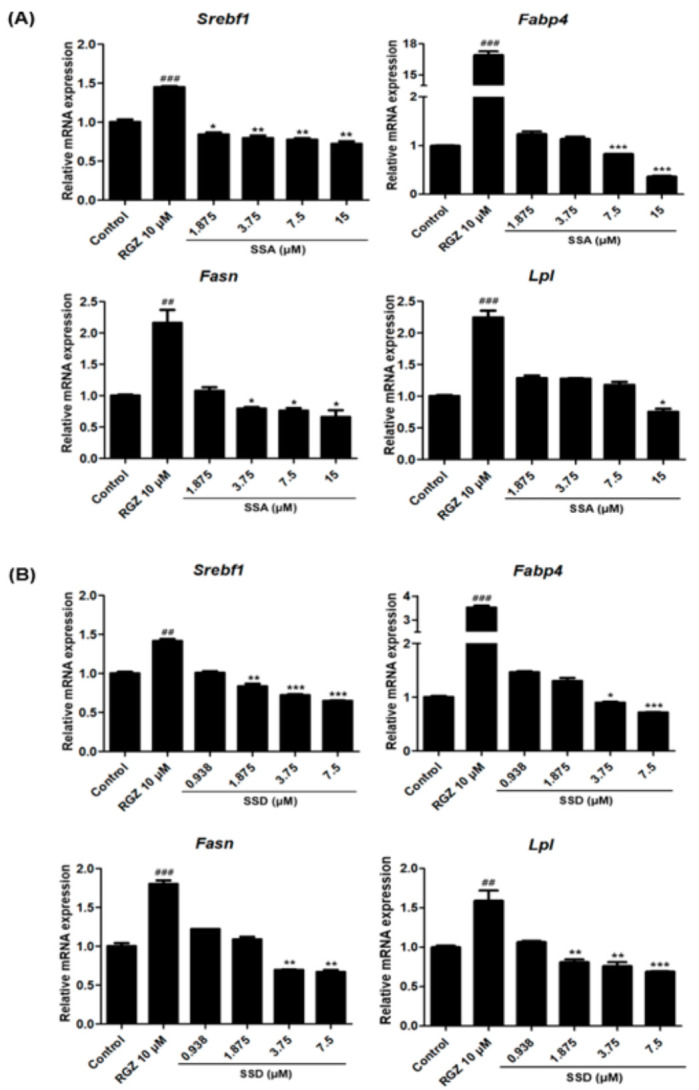
Effect of SSA (**A**) and SSD (**B**) on the mRNA expression levels of adipogenic transcriptional factors in 3T3-L1 cells as measured by quantitative PCR. Results are shown as relative of control (0.5% DMSO) values and RGZ (rosiglitazone) was used as a positive control for this experiment. *Gapdh* was used as a house keeping gene. Target gene mRNA levels were normalized to *Gapdh* using the 2-ΔΔ Ct method. Results are mean ± standard error of the mean (SEM) of three independent experiments. * *p* < 0.05, ** *p* < 0.01, *** *p* < 0.001, ## *p* < 0.01, ### *p* < 0.001, compared with control, #: increased compared to control, *: decreased compared to control.

To further investigate the mechanisms of SSA and SSD suppression of adipogenesis, we confirmed the mRNA expressions of adipocyte-specific transcription factors by qRT-PCR. As shown in Figure 4, the mRNA expression of Srebf1 was dose-dependently suppressed in SSA and SSD treated 3T3-L1 adipocytes. Moreover, these compounds suppressed the mRNA expressions of *Fabp*, *Fasn*, and *Lpl*, which are associated with lipogenesis and adipogenesis.

### 2.5. Effects of SSA and SSD on the Expression Levels of Adiponectin in 3T3-L1 Cells

**Figure 5:**
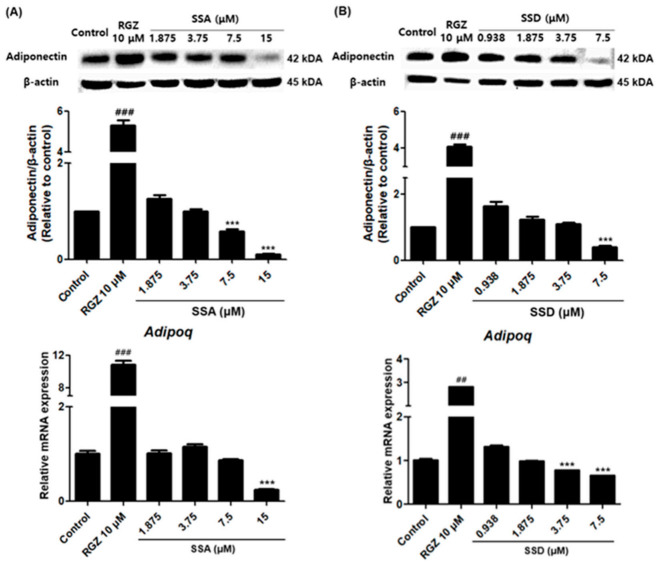
Effect of SSA (**A**) and SSD (**B**) on the expression protein and mRNA levels of adiponectin in 3T3-L1 cells as measured by Western blot and quantitative real-time PCR, respectively. Results are shown as a relative of control (0.5% DMSO) values and RGZ (rosiglitazone) was used as a positive control for this experiment. β-actin was used as a loading control and *Gapdh* was used as a house keeping gene. Target gene mRNA levels were normalized to *Gapdh* using the 2-ΔΔ Ct method. Data are mean ± standard error of the mean (SEM) of three independent experiments. Results are mean ± standard error of the mean (SEM) of triplicate experiments. *** *p* < 0.001, ## *p* < 0.01, ### *p* < 0.001, compared with control, #: increased compared to control, *: decreased compared to control.

We measured the effects of SSA and SSD on the expression of adiponectin, which is known to increase during the 3T3-L1 differentiation process. The highest concentrations of SSA and SSD significantly reduced the protein expression level of adiponectin by approximately 89% and 60%, respectively, relative to the control group (*p* < 0.001) (Figure 5). In addition, these two constituents had similar dose-dependent effects on the mRNA levels of *adipoq*.

### 2.6. Effects of SSA and SSD on the AMPK Signaling Pathway

**Figure 6:**
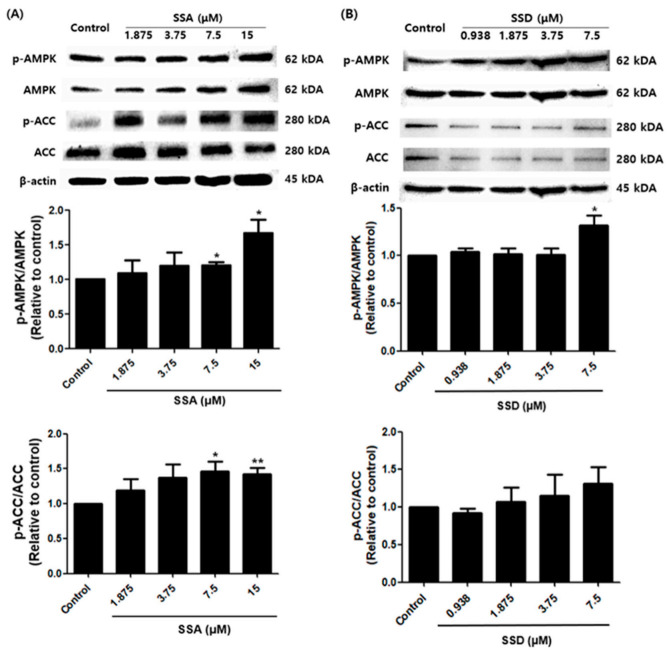
Effect of SSA (**A**) and SSD (**B**) on adipogenesis is mediated by AMPK signaling pathway as examined by Western blot analysis. The graphs show the band intensity ratio of the phosphorylated form by the total protein expression of AMPK and ACC. Results are shown as a relative of control (0.5% DMSO) values for this experiment. β-actin was used as a loading control. Data are mean ± standard error of the mean (SEM) of three independent experiments. * *p* < 0.05, ** *p* < 0.01, compared with control, *: increased compared to control.

To obtain insight into the action mechanism of SSA- and SSD-linked adipogenesis inhibition, crucial AMPK pathway protein levels were analyzed. AMPK and its substrate ACC are key regulators of the adipocyte differentiation process and adipogenesis. Interestingly, SSA significantly increased the phosphorylation of AMPK and its substrate ACC compared with controls. In contrast, SSD enhanced the phosphorylation of AMPK compared with controls, although the phosphorylation of ACC was not statistically significant (Figure 6).

### 2.7. Effects of SSA and SSD on the MAPK Signaling Pathway

**Figure 7:**
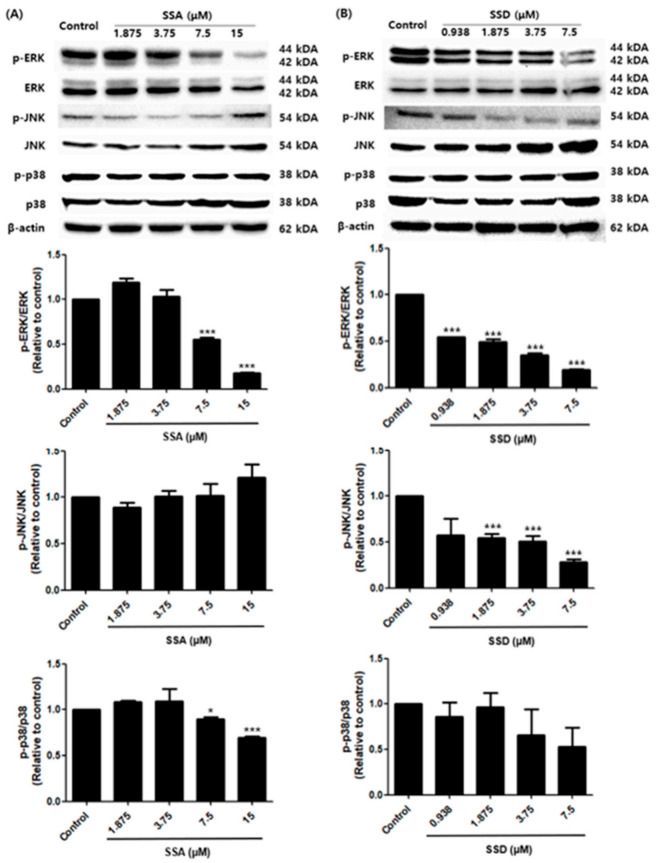
Effect of SSA (**A**) and SSD (**B**) on adipogenesis is mediated by MAPK signaling pathway as examined by Western blot analysis. The relative intensity of each band of the phosphorylated ERK, JNK, and p38 after normalization for the levels in the total forms is shown, and β-actin was used as a loading control. Results are shown as a relative of control (0.5% DMSO) values for this experiment. Data are mean ± standard error of the mean (SEM) of three independent experiments. * *p* < 0.05, *** *p* < 0.001, compared with control, *: decreased compared to control.

Next, to investigate the effect of SSA and SSD on the MAPK signaling pathways in 3T3-L1 cells, we measured the phosphorylation of ERK, JNK, and p38, which have a regulatory role in the early stage of adipogenesis. As shown in Figure 7, treatment with SSA tended to decrease the phosphorylation of ERK and p38 in a dose-dependent manner. However, we did not confirm any inhibitory effect of SSA on the phosphorylation of JNK. In contrast, SSD downregulated the phosphorylation of ERK and JNK compared with controls (*p* < 0.001). The phosphorylation of p38 showed a tendency to decrease in a concentration-dependent manner, but this was not statistically significant. Thus, it suggests that SSA and SSD can downregulate adipogenesis through the MAPK signaling pathway.

## 3. Discussion

Obesity causes or exacerbates many health problems independently or in connection with other diseases such as type 2 diabetes mellitus, coronary heart disease, respiratory complications, and osteoarthritis, and research has been conducted in countries around the world with higher interest than ever before [22]. Adipocytes are the most common cell type in adipose depots and occur in white and brown fat [23]. Brown adipocytes are mainly responsible for non-shivering thermogenesis in response to low temperature stress or β-adrenergic stimulus, whereas white adipocytes store excess energy as triglycerides via adipogenesis and use triglycerides during food deprivation via lipolysis [24]. Excessive nutrients can increase the cell size of existing adipocytes (hypertrophy) or increase their number through the differentiation of new adipocytes (hyperplasia), leading to the abnormal expansion of white adipocytes associated with obesity [25,26]. Thus, controlling the size and number of adipocytes could be a possible therapeutic approach for obesity, and identifying potential adipogenic molecular targets that are susceptible to regulation by external factors can utilize adipogenesis modulation to control obesity [6].

In this study, we treated cells with SSA or SSD, triterpenoid saponins derived from *Radix Bupleuri* during the adipogenesis process. As a result, it was confirmed by Oil Red O staining that SSA and SSD suppressed lipid accumulation without decreasing cell viability in a dose-dependent manner. Numerous studies have reported that various natural triterpenoid saponins inhibit adipogenesis and downregulate the cellular lipid accumulation of ginsenoside Rb1 [27], ginsenoside Rh1 [28], ursolic acid [29], and kudinoside-D [30].

The adipogenesis process is tightly modulated by transcription cascades and signaling pathways. In the early stages of differentiation, C/EBPβ/δ is transiently high. It stimulates PPARγ and C/EBPα, which are the key transcription factors for adipogenesis in the intermediate stage of adipogenesis [31,32]. Our finding revealed that SSA and SSD significantly reduced PPARγ and C/EBPα, which are essential for adipogenesis, at the protein and mRNA levels. During the terminal stage of differentiation, PPARγ and C/EBPα promote the induction of several adipocyte-specific genes, including *Fabp4*, *Fasn*, and *Lpl* [33]. Fabp4, commonly known as adipocyte protein 2 (aP2), is a predominant facilitator of lipid uptake, transport, and metabolism [34]. FAS is important for adipogenesis as the major enzyme involved in long-chain saturated fatty acid synthesis in 3T3-L1 adipocytes [35]. Another adipocyte marker, LPL, is responsible for the hydrolysis of triglycerides and is a key enzyme for lipoprotein metabolism [36]. SREBPs are transcription factors encoded by the *Srebf1* and *Srebf2* genes, and they have three isoforms (SREBP-1a, -1c, and -2). Of these, SREBP-1c serves as a central hub in lipid metabolism and is the predominant isoform expressed in most tissues in mice and humans, with especially high levels in the liver, white adipose tissue, skeletal muscle, adrenal gland, and brain [37]. According to our study, SSA and SSD downregulated these adipocyte-specific targets at the mRNA level in a dose-dependent manner.

Interestingly, SSA and SSD significantly decreased the expression of adiponectin. These data suggest a new role for adiponectin in adipocyte biology. Several clinical studies have indicated that circulating adiponectin levels are decreased in patients with insulin resistance, type 2 diabetes mellitus, and obesity [38,39]. However, in vivo studies have reported that adding adiponectin to cloned stromal preadipocytes inhibited the differentiation of these cells into fat cells, although they also observed no such inhibition in 3T3-L1 adipocytes [40]. In addition, the overexpression of adiponectin enhanced 3T3-L1 fibroblast proliferation, accelerated adipocyte differentiation, and, in fully differentiated adipocytes, augmented both lipid accumulation and insulin-responsive glucose transport [41]. Indeed, the expression of adiponectin was not observed in the early and intermediate stages of our differentiation process. However, the expression of adiponectin was dramatically increased at the terminal stage of differentiation (data not shown). In addition, the expression of adiponectin was significantly increased in the rosiglitazone treatment group, which promoted differentiation compared with the control group. Therefore, the results of this experiment show that adiponectin reduction by SSA and SSD is due to their anti-adipogenic effects.

AMPK acts as a central modulator of energy sensing and homeostasis [11]. Phosphorylated AMPK promotes catabolic pathways such as fatty acid oxidation and inhibits energy consumption pathways such as fatty acid synthesis [42]. ACC, one of the target molecules of AMPK, is a crucial rate-regulating enzyme for fatty acid metabolism [43]. Our findings showed that SSA and SSD enhanced the phosphorylation of AMPK and ACC during 3T3-L1 differentiation.

The ERK, JNK, and p38 mitogen-activated protein kinases are intracellular signaling pathways that play a pivotal role in the processes of proliferation and differentiation [44]. The activation of ERK is essential for inducing adipogenesis and the necessary differentiation process called mitotic clonal expansion [45]. Adipocyte differentiation was promoted by the activation of several kinases, specifically PI3K kinase, activating p38, resulting in adipocyte synthesis [11]. Both compounds reduced the phosphorylation of ERK and p38 in a dose-dependent manner. However, the effect of SSD on the phosphorylation of p38 was not statistically significant. Furthermore, in the case of the phosphorylation of JNK, SSD showed a decreasing effect, whereas SSA showed a slight increase without statistical significance. Both the positive and negative regulation of JNK in the process of adipocyte differentiation is suggested [46]. According to a recent report, JNK can contribute to the inhibition of adipogenesis by enhancing the activation of the inflammatory signaling pathway as well as nuclear factor kappa light chain enhancer of activated B cells (NF-κB) under the influence of pro-inflammatory molecules.

It is necessary to determine whether the effect of SSA and SSD on adipogenesis downregulation is altered with specific inhibitors or stimulators that affect the activation of AMPK (e.g., 5-aminoimidazole-4-carboxamide ribonucleoside (AICAR), dorsomorphin) and MAPK (e.g., PD98059, SB203580), to clarify the direct link between adipogenesis process and two signaling pathways. The lack of these experiments is a limitation of our study, and further studies will enable to reveal a clear mechanism for the anti-adipogenic effects of SSA and SSD.

## 4. Materials and Methods

### 4.1. Chemicals and Reagents

Saikosaponin A and D were purchased from Chengdu Biopurify Phytochemicals (Chengdu, China). The 3T3-L1 adipocytes were provided by the Korean Cell Line Bank (Seoul, Korea). Dulbecco’s modified Eagle’s medium, High glucose (DMEM/HG), newborn calf serum (NBCS), fetal bovine serum (FBS), and penicillin–streptomycin solution (P-S) were purchased from Thermo Fisher Scientific (Waltham, MA, USA). Phosphate buffered saline (PBS), 10% formalin, Oil Red O, insulin, and dexamethasone were obtained from Sigma-Aldrich (St. Louis, MO, USA). Dimethyl sulfoxide (DMSO) and 3-(4,5-dimethylthiazol-2-yl)-2,5-diphenyltetrazolium bromide (MTT) were supplied by Glentham Life Sciences (Corsham, UK). Isopropanol, 3-isobutyl-1-methylxanthine (IBMX), goat anti-rabbit IgG (AP132P, 1:4000), and goat anti-mouse IgG (AP124P, 1:4000) were obtained from Millipore (Burlington, MA, USA). Rosiglitazone (RGZ) was purchased from Cayman Chemical (Ann Arbor, MI, USA). Antibodies against PPARγ (2435S, 1:1000), C/EBPα (8178, 1:1000), adiponectin (2789, 1:2000), AMP-activated protein kinase (AMPK, 2532, 1:1000), phospho-AMPK (*p*-AMPK; 2531, 1:1000), acetyl-CoA carboxylase (ACC; 3662, 1:1000), phospho-ACC (*p*-ACC; 3661, 1:1000), extracellular signal-related kinase (ERK; 4695, 1:1000), phospho-ERK (*p*-ERK, 4370, 1:1000), p38 (8690, 1:1000), phospho-p38 (*p*-p38; 4511, 1:1000) and β-actin (4967, 1:1000) were from Cell Signaling Technology (Danvers, MA, USA), and c-Jun-N-terminal kinase (JNK; sc-7345, 1:250) and phospho-JNK (*p*-JNK; sc-293136, 1:250) were provided by Santa Cruz Biotechnology (Santa Cruz, CA, USA).

### 4.2. Cell Culture and Differentiation

**Figure 8:**
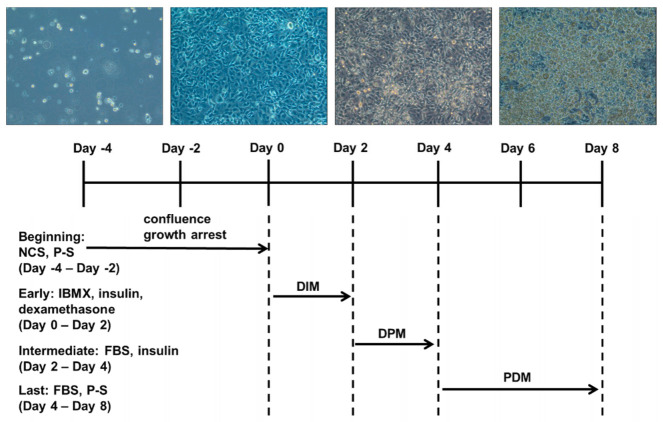
3T3-L1 cell differentiation processes.

The 3T3-L1 preadipocytes were cultured in DMEM/HG supplemented with 10% NBCS, 1% P-S at 37 °C in a humidified 5% CO_2_ atmosphere. Two days later, cells were maintained in the same medium until growth arrest (differentiation day 0). After full confluence, differentiation was induced in differentiation initiation medium (DIM) comprising DMEM/HG containing 10% FBS, 0.5 mM 3-isobutyl-1-methylxanthine (IBMX), 10 μg/mL insulin, and 1 μM dexamethasone. The differentiation medium was withdrawn 2 days later (differentiation day 2) and switched to differentiation progression medium (DPM) comprising DMEM/HG supplemented with 10% FBS and 10 μg/mL insulin. After 2 days in the insulin containing medium (differentiation day 4), cells were cultured in post-differentiation medium (PDM) containing 10% FBS and 1% P-S which was changed every 2 days until day 8 (Figure 8).

### 4.3. Cell Viability

Cell viability was assessed by MTT assay. The 3T3-L1 cells were grown in 96-well plates at a density of 1 × 10^4^ cells/well comprising DMEM/HG supplemented with 10% NBCS and 1% P-S, and incubated for 24 h until full confluence. After reaching confluency, different concentrations (0–100 μM) of saikosaponins were added to the medium. Cells treated with 0.5% DMSO were used as controls. After 48 h, 5 mg/mL MTT 20 μL was added to the sample medium in each well for 2 h incubation. The solution was removed and DMSO (100 μL/well) was added for 10 min to dissolve the formazan crystals. In this assay, the ability of metabolically active cells to convert MTT into a blue formazan product was measured and its absorbance was recorded at 540 nm using a microplate reader (Bio-Rad, Hercules CA, USA). Results were expressed as a percentage of viable cells relative to control. The experiments were performed in triplicate.

### 4.4. Oil Red O Staining and Quantification

Lipid accumulation was assessed by Oil Red O staining. The differentiated 3T3-L1 cells were washed twice with PBS at a pH of 7.4 and fixed in 10% formalin for 30 min at room temperature. After removing formalin, the fixed cells were washed twice with distilled water and stained with Oil Red O working solution (60% filtered Oil Red O stock solution) for 30 min at room temperature. Next, the staining solution was removed, and the cells were washed thrice with distilled water. Microscope images were taken to visualize lipid droplet staining in differentiated cells. To quantify the cellular lipid amount, isopropanol was dispensed into the cells and shaken at room temperature for 30 min. Thereafter, Oil Red O in the supernatant was measured at 520 nm using a microplate reader (Bio-Rad).

### 4.5. Western Blot Analysis

The 3T3-L1 cells differentiated for 8 days in the absence or presence of saikosaponins were washed with ice-cold PBS, scraped into a pre-cooled 1.5 mL Eppendorf tube, and centrifuged. Then, the resulting cell pellets were lysed in ice-cold 1× radioimmunoprecipitation assay (RIPA) buffer consisting of 50 mM Tris HCl, 150 mM NaCl, 1.0% NP-40, 0.5% sodium deoxycholate, 1.0 mM EDTA, 0.1% SDS, 0.001% sodium azide at a pH of 7.4, protease, and a phosphatase inhibitor cocktail (Quartett, Berlin, Germany) and left on ice for 30 min. After the lysates were centrifuged, the supernatant was used as the whole cell lysate and the protein concentration was determined using the Bradford assay. Each protein sample (40 µg) was separated by 10% sodium dodecyl sulfate-polyacrylamide gel electrophoresis (SDS-PAGE) and transferred to a polyvinylidene difluoride (PVDF) filter membrane (Bio-Rad). The membranes were blocked for 1 h at room temperature using 5% skim milk in Tris-buffered saline and 0.1% Tween 20 (TBS-T). The membranes were washed with TBS-T 3 times for 5 min each time, and subsequently incubated overnight at 4 °C with the primary antibody. After washing, they were incubated with an appropriate secondary antibody at room temperature for 1 h and then the membranes were washed again with TBS-T 5 times for 5 min each time. Secondary antibody binding was visualized using a Chemi Doc XRS+ imaging system (Bio-Rad). The intensity of each immunoreactive band was performed using the Image Lab Software Program (Bio-Rad).

### 4.6. Quantitative Real-Time PCR

Total RNA was isolated from differentiated 3T3-L1 cells using NucleoZOL (Macherey-Nagel, Düren, Germany) according to the manufacturer’s instructions. The purity and concentration of total RNA were determined using Nanodrop (Thermo Scientific, Wilmington, DE, USA). Then, mRNA purification of total RNA was performed using the NucleoTrap mRNA purification kit (Macherey-Nagel) and full-length cDNAs were synthesized from mRNA by a ReverTraAce^®^ qPCR RT kit (Toyobo, Osaka, Japan). Quantitative real-time PCR was performed using the CFX384 Touch Real-Time PCR Detection System (Bio-Rad) according to the manufacturer’s instructions, and gene expression was measured using SYBR-green (Bio-Rad) and gene-specific primer sets. The sequences of the primers used in this experiment are listed in Table 1. Reactions were performed under the following conditions: one cycle at 95 °C for 3 min, followed by 39 cycles at 95 °C for 10 s and 60.5 °C for 30 s, and then one cycle at 95 °C for 10 s, one cycle at 65 °C for 0.05 s and 95 °C for 0.5 s. The glyceraldehyde 3-phosphate dehydrogenase (*Gapdh*) was used as a house keeping gene. Target gene mRNA levels were normalized to *Gapdh* using the 2-ΔΔ Ct method.

### 4.7. Statistical Analysis

All data were presented as the mean ± standard error of the mean (SEM) of triplicates. Differences between mean values for control and samples for each concentration were analyzed by the Student’s *t*-test. The *p* values < 0.05 were considered statistically significant.

## 5. Conclusions

**Figure 9:**
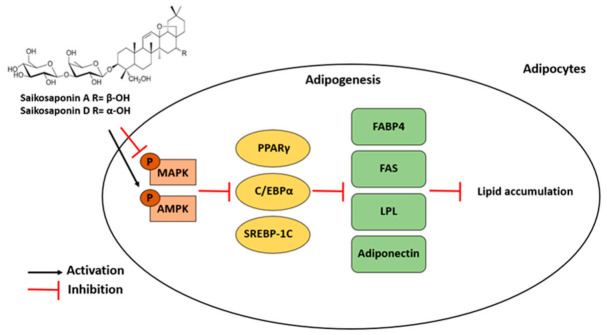
Suggested molecular mechanism for anti-adipogenic effect of saikosaponin A (SSA) and saikosaponin D (SSD) in 3T3-L1 adipocytes. Black arrows indicate activation and red arrows refer to inhibition.

In summary, the experimental results indicated that the natural triterpenoid saponins SSA and SSD from *Radix Bupleuri* significantly suppressed lipid accumulation in 3T3-L1 cells. The inhibitory effects of SSA and SSD on adipogenesis were mediated by the regulation of adipocyte-specific targets via the AMPK and MAPK signaling pathways (Figure 9). Therefore, SSA and SSD are promising natural products for the prevention of obesity and metabolic disorders.

## Figures and Tables

**Table 1:** Oligonucleotide sequence used in quantitative RT-PCR.

Name	Sequence of Primers (5′→3′)	Annealing Temp (°C)
*Pparg*	F: CAAGAATACCAAAGTGCGATCAA R: GAGCTGGGTCTTTTCAGAATAATAAG	58.4
*Cebpa*	F: AGGTGCTGGAGTTGACCAGT R: CAGCCTAGAGATCCAGCGAC	60.5
*Srebf1*	F: GCTTAGCCTCTACACCAACTGGC R: ACAGACTGGTACGGGCCACAAG	65.9
*Adipoq*	F: AGCCTGGAGAAGCCGCTTAT R: TTGCAGTAGAACTTGCCAGTGC	60.5
*Fabp4*	F: AAGACAGCTCCTCCTCGAAGGTT R: TGACCAAATCCCCATTTACGC	64.7
*Fasn*	F: TTGCTGGCACTACAGAATGC R: AACAGCCTCAGAGCGACAAT	58.4
*Lpl*	F: AGG ACC CCT GAA GAC ACA GCT R: TGT ACA GGG CGG CCA CAA GT	63.3
*Gapdh*	F: TTGTTGCCATCAACGACCCC R: GCCGTTGAATTTGCCGTGAG	60.5

## Data Availability

The data presented in this study are available on request from the corresponding author.
